# Entry Without Expression: Internalisation Does Not Predict mRNA Translation for Targeted Lipid Nanoparticles

**DOI:** 10.1002/smsc.70313

**Published:** 2026-05-21

**Authors:** Cameron H. Smyth, Lara M. Mollé, Victoria McLeod, Moore Z. Chen, Angus P. R. Johnston

**Affiliations:** ^1^ Drug Delivery, Disposition and Dynamics Monash Institute of Pharmaceutical Sciences Monash University Parkville Victoria Australia

**Keywords:** endosomal escape, flow cytometry, internalisation, lipid nanoparticles, nucleic acid delivery

## Abstract

Lipid nanoparticle (LNP) delivery systems offer efficient encapsulation and protection of mRNA, making them a popular platform in nucleic acid therapeutics, including mRNA vaccines. Conventional LNPs rely on non‐specific cellular interactions and internalisation which can result in poor delivery to target cells and increased off‐target delivery. Surface functionalization of LNPs with antibodies can enhance target cell accumulation, reducing off target effects. However, selecting antibody‐receptor pairs based on abundance does not result in optimal delivery. Here, we present a novel approach to quantify internalisation and mRNA delivery efficiency of antibody‐functionalised LNP/mRNA in primary human T and B cells. By targeting LNPs to a range of clinically relevant T and B cell receptors, we demonstrate that LNP internalisation does not necessarily predict successful mRNA delivery. Our findings highlight that receptor mediated internalisation can improve targeted delivery of LNPs, however favourable receptor‐specific post‐internalisation trafficking of LNP/mRNA is critical to successful cytosolic mRNA release and translation.

## Graphical abstract

Internalisation of mRNA–lipid nanoparticles (LNPs) into cells does not predict the level of protein expression. We used an internalisation sensor to quantify association, uptake, and mRNA translation of targeted LNPs in human T and B cells. Antibody targeting enhanced cell‐specific association, however receptor‐dependent intracellular processing emerged as the key determinant of functional mRNA delivery.

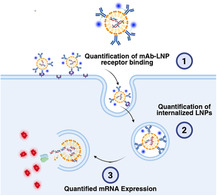

## 1 Introduction

Lipid nanoparticle (LNP) delivery systems have transformed the delivery of nucleic acid therapeutics [1, 2]. They overcome the rapid degradation of RNA that occurs when it is delivered in vivo and offer a scalable platform for the production of nucleic acid therapeutics. The field advanced rapidly during the COVID‐19 pandemic [3, 4, 5], with the successful development and deployment of LNP‐encapsulated mRNA vaccines. However, despite their success, most LNPs still rely on passive distribution and inefficient uptake mechanisms, which present key challenges in their further development [6].

To overcome the limitations of passive delivery, targeting groups such as antibodies can be attached onto LNPs, which increases association to specific cells. A popular approach is to couple antibodies to LNPs via the cysteine or lysine residues [7, 8], however this approach requires time consuming purification of each targeted LNP preparation to remove unbound antibody. To address this, our group developed a versatile site‐ and orientation‐specific conjugation method to attach monoclonal antibodies (mAbs) to the surface of LNPs [9]. This method enables the quantitative capture of antibodies onto the surface of LNPs without the need for further purification. This enables us to generate an array of LNPs functionalised with different mAbs and allows high‐throughput screening of receptors for optimal binding and activity. Furthermore, our capture strategy also improves protein expression by eightfold compared to conventional random antibody–LNP conjugation methods.

Identification of an optimal receptor for targeted delivery is not determined solely by surface abundance or cellular specificity. Our previous studies have shown that receptor expression on the cell surface is not necessarily correlated with productive mRNA delivery. Internalisation of the LNP (and receptor) plays a critical role in determining whether the mRNA cargo reaches the cytosol for translation. Consequently, understanding LNP internalisation is essential for the rational design of effective targeted mRNA–LNP delivery systems.

**FIGURE 1:**
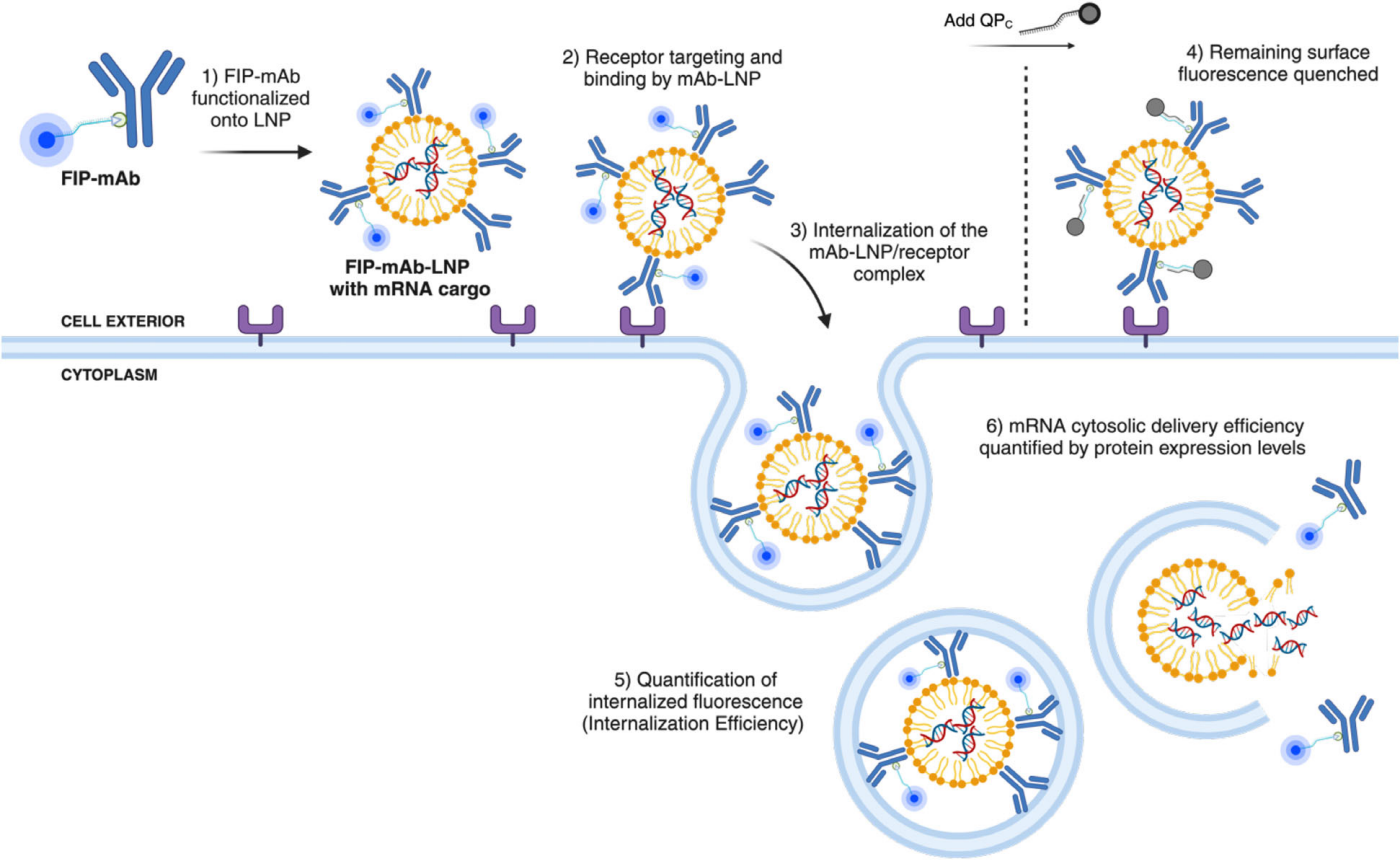
Quantifying FIP‐mAb functionalised LNP internalisation and mRNA delivery efficiency via resulting protein expression. Using FIP‐mAbs functionalised onto LNPs to simultaneously quantify internalisation and mRNA delivery using the SHIP assay. (1) Generated FIP‐mAbs are functionalised onto LNPs via surface present anti‐Fc nanobody. (2) Sensor incubation with cells promotes receptor binding and (3) internalisation of the LNPs occurs through the target receptor. (4) Quenching of remaining surface signal occurs following quencher addition. (5) Flow cytometry is used to measure internalised Cy5‐FIP‐mAb‐LNPs for quantification of internalisation and (6) the efficiency of mRNA cargo delivery by surrogate measurement of subsequent protein expression levels. Created in Biorender Molle, L.

To address this, we have applied our previously developed internalisation assay (specific hybridisation internalisation probe – SHIP, Figure 1) [10] which measures signal of fluorescent internalisation probe (FIP) to understand the important roles that cell association, cell uptake, and internalisation efficiency have on the final delivery of active mRNA. In this study we investigated a range of mAb‐LNP/mRNA targeted to clinically relevant T and B lymphocyte receptors for transfection of specific cell populations of primary human peripheral blood mononuclear cells (PBMCs) ex vivo. Interestingly, we found that association and internalisation on mAb‐LNP/mRNA did not always translate to active mRNA delivery. This strongly suggests that the trafficking pathways of the targeted receptors plays a critical role in the successful delivery of active mRNA.

## 2 Results and Discussion

### 2.1 Development of FIP‐mAb‐LNPs for Internalisation Screening

The SHIP assay is a method we previously developed for quantifying the uptake of proteins and nanoparticles into cells [10]. It uses a FIP, which is a Cy5‐labelled single‐stranded DNA oligonucleotide conjugated to a mAb of interest [10]. When a complementary, membrane impermeable quencher strand (QP_C_) is added to cells with internalised protein, surface bound FIP is quenched, while the internalised protein remains fluorescent. In addition to measuring the total amount of internalised protein, we can also calculate internalisation efficiency, which is defined as the ratio of internalised to total cell‐associated material. The SHIP assay does not interfere with the measurement of nucleic acid activity (protein expression), allowing active delivery of mRNA to be measured alongside association, uptake and internalisation efficiency. Understanding these critical parameters is important for screening of mAb/receptor behaviour for optimised mRNA delivery and expression.

**FIGURE 2:**
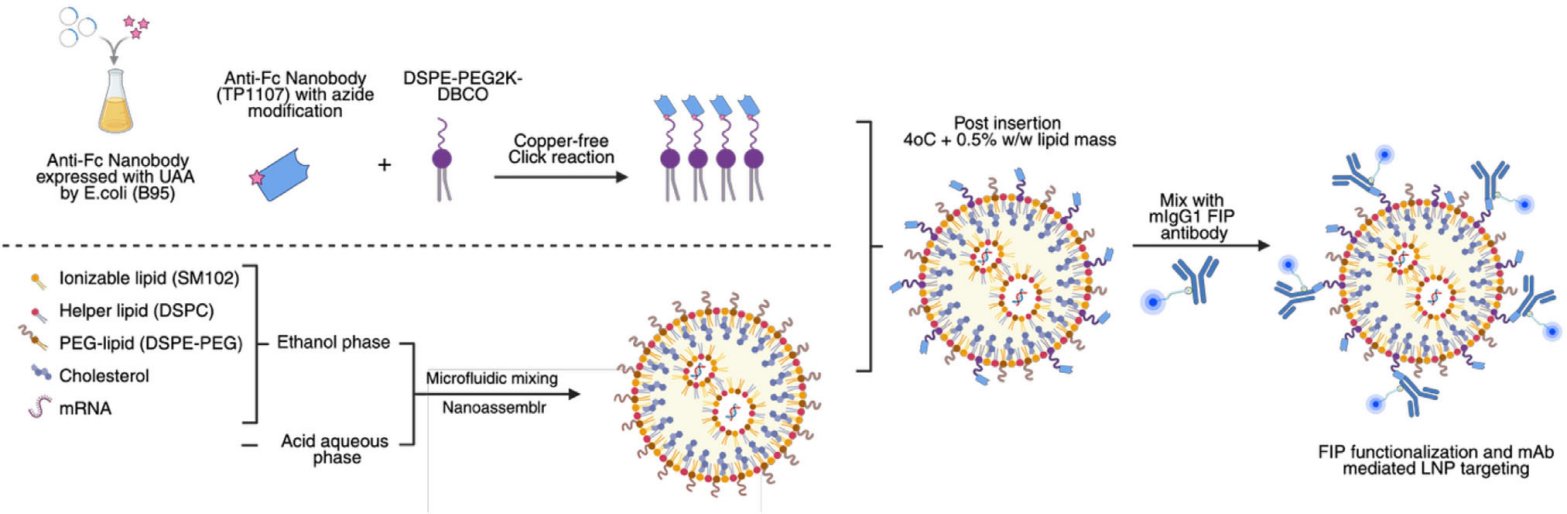
LNPs that capture antibodies using an anti‐FcNb can be used to generate FIP‐mAb‐targeted LNPs for mRNA delivery and measurement of internalisation. The schematic outlines the process of anti‐FcNb insertion and FIP‐antibody capture onto the LNP surface to form FIP‐mAb‐targeted lipid nanoparticles (LNPs). Figure adapted from Chen et al. [9].

Attaching mAbs to LNPs using our versatile anti‐Fc nanobody capture system provides a high‐throughput method for screening of mAb‐LNPs. To incorporate SHIP assay measurements into this system, targeting mAbs were functionalised with FIP using click chemistry, and then subsequently attached to the LNP surface via Fc‐binding using our antibody capture system (Figure 2) [9]. Binding, uptake and internalisation efficiency of FIP‐mAb‐mRNA/LNPs can then be determined using the SHIP assay (Figure 1), while simultaneously measuring protein expression from the cargo mRNA.

Five mAbs that target T and B cell receptors were investigated. Two mAbs targeting T cell specific receptors (CD3 and CD7), two mAbs targeting B cell specific receptors (CD20 and CD22) and one mAb targeting a broader lymphocyte receptor (CD45) were chosen. An additional isotype control (mIgG1) was also included to investigate non‐specific binding and expression in the cells. Successful click‐conjugation of FIP to the targeting mAbs was confirmed by the presence of the fluorescent band at ∼150 kDa (Figure S1). Degree of labelling was calculated for all FIP‐mAbs (Table S1) and were applied when calculating association and internalisation efficiency, as outlined in the methods. Functionalisation of the LNPs with each of the FIP‐mAbs was achieved by adding the antibody to the anti‐FcNb‐LNPs at a twofold excess of anti‐FcNb to mAb, which ensured efficient binding of the FIP‐mAb binding by the anti‐FcNb‐LNPs (Figure S2). The anti‐FcNb conjugation method allows for a single anti‐FcNb formulation to be characterised (size, encapsulation efficiency and mRNA concentration – Table S2) before being incubated with the desired mAbs. After mAb incubation, no further purification or processing is required before incubation with cells. This approach overcomes the need to characterise individual mAb‐LNP batches and provides consistent mRNA formulations across each unique mAb‐LNP.

LNPs were measured by NTA (NanoSight) and formed a single population with a diameter of 101 ± 3.1 nm (*n* = 3) (Table S2). A single population is maintained following the addition of a FIP‐mAb onto the LNP surface, demonstrated with the FIP‐mIgG1 isotype control, saw a small increase in diameter compared to the untargeted LNPs (108 ± 8.3 nm, *n* = 3).

### 2.2 Investigating Internalisation of FIP‐mAb‐LNPs Targeting Prominent B and T Cell Markers

FIP‐mAb‐LNP/receptor internalisation (targeting CD3, CD7, CD20, CD22 and CD45, plus the isotype IgG control) was investigated after 4‐h incubation with PBMCs isolated from healthy donors. The targeted LNPs were incubated with PBMCs, after which phenotyping antibodies were added to separate cell populations into CD4+ T cells, CD8+ T cells and B cells (Figure S3). Association, uptake and receptor internalisation efficiency were calculated using the generated Cy5 signal by the FIP‐mAb‐LNPs, as outlined in the experimental details. Protein expression levels were quantified using detection of fluorescent red protein, mScarlet.

Internalisation efficiency is calculated by ratioing uptake to total cell association, therefore antibodies with very low levels of association can lead to a large amount of error (through division by a small number). Therefore, a threshold for calculating the internalisation efficiency of a receptor was determined, whereby the total association of the FIP‐mAb‐LNP must be significantly higher (*p* < 0.05) than the MFI generated by the untreated cells. MFI values that are not significantly higher than untreated cells suggest that non‐specific interactions are occurring rather than specific binding of the target receptor. Incubation with FIP‐mAb‐LNPs was limited to 4 h to capture rapid association and internalisation behaviour, rather than non‐specific membrane turnover, which may obscure receptor specific internalisation trends.

### 2.3 B Cells Show Low Reporter Protein Expression With CD22 Targeted LNPs, Despite Having Highly Efficient Internalisation

B lymphocytes play a pivotal role in adaptive immunity through the generation of humoral responses. Given that approximately 90% of lymphomas originate from B cells, their surface receptors have emerged as particularly attractive targets for therapeutic intervention [11]. CD20 and CD22 are prominent B cell specific receptors with clinical mAb‐based therapeutic successes rituximab for treatment of Non‐Hodgkin's lymphoma, and inotuzumab ozogamicin for relapsed/refractory B cell acute lymphoblastic leukaemia [12, 13].

**FIGURE 3:**
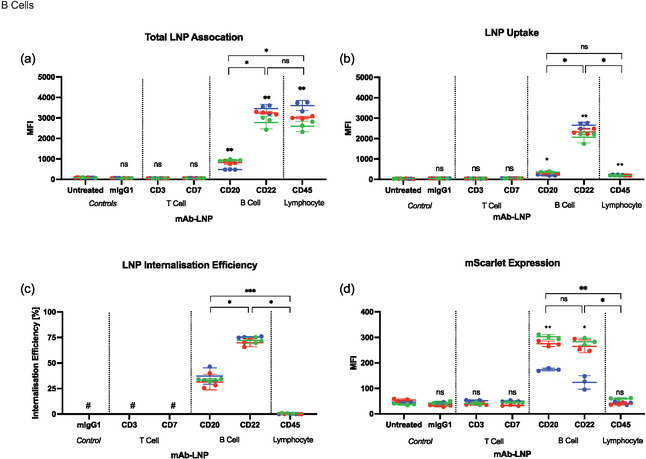
Measuring mAb‐LNP internalisation and delivery in B cells after 4 h. SHIP assay outputs result in quantification of (a) total association (MFI), (b) uptake of mAb‐LNPs (MFI) and (c) internalisation efficiency (%) of the receptor when bound by the mAb‐LNP. (d) represents the mScarlet protein expression. Cy5‐FIP and mScarlet fluorescence were analysed by flow cytometry at 649 and 580 nm, respectively. Uptake of the mAb‐staple sensor through each receptor was measured following the addition of quencher DNA (500 nM). Statistical analysis was performed using 2‐way ANOVA with Dunnett's test. Each mAb‐LNP was compared to the untreated control. p > 0.05 is not significant (ns), * indicates p < 0.05, ** indicates p < 0.01, *** indicates p < 0.001 and **** indicates p < 0.0001. Significance shown is representative of the mean calculated p value of the donors. # signifies receptors that did not have sufficient association compared to mIgG1 isotype control for internalisation to be calculated. Each colour is representative of a single donor (with technical replicates). Data is mean ± SD (n = 3 individual donors and 3 technical replicates).

In B cells, after 4 h, mAb‐LNPs targeting CD22 (MFI 3158 ± 355) and CD45 (MFI 3071 ± 478) showed significantly higher association than LNPs targeting CD20 (MFI 746 ± 207) (Figure 3a). A similar trend for association/expression was observed after 24 h (Figure S5). As expected, all three B cell targeted LNPs showed significantly high association to B cells compared to untreated cells and LNPs that target the T cell receptors (CD3 and CD7) (MFI < 100; Figure 3a).

Interestingly, uptake of LNPs into B cells varied significantly from the association profile (Figure 3b). CD22 targeted LNPs showed significantly higher uptake (MFI 2351 ± 310) than both CD20 (MFI 287 ± 73) and CD45 targeted LNPs (MFI 202 ± 22; Figure 3b). This corresponds to high internalisation efficiency (>70%) of CD22 targeted LNPs (Figure 3c), whereas the internalisation efficiency of CD20 targeted LNPs was ∼34% and CD45 targeted LNPs < 1% (Figure 3c). CD20 targeted LNPs showed ∼20% increased internalisation efficiency compared to the antibody itself (based on a previous internalisation study using the same antibody), whereas CD45 targeted LNPs showed similar internalisation efficiency to the free antibody [14]. CD22 targeted LNPs, however, exhibited ∼15% lower internalisation than the antibody itself, suggesting an interference of internalising pathways with conjugated LNPs [14].

Interestingly, despite CD22 targeted LNPs having more efficient internalisation than CD20 targeted LNPs, the resulting mScarlet expression was similar (MFI 223 ± 78 vs MFI 250 ± 59; Figure 3d). Furthermore, CD45 targeted LNPs had no significant mScarlet expression despite having similar total uptake to CD20 targeted LNPs (Figure 3b). These data suggest that receptor trafficking following internalisation plays an important role in cytosolic delivery of the mRNA cargo and should be considered when selecting a receptor for targeting.

**FIGURE 4:**
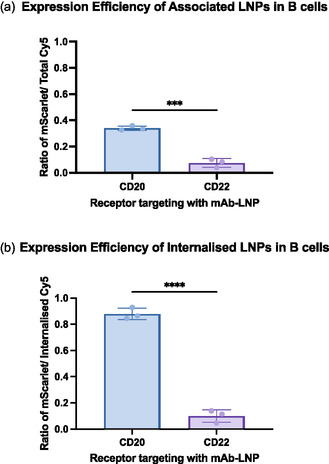
mScarlet expression efficiency of mAb‐LNPs for B cell receptors calculated with total association and uptake. Ratio of MFI (mScarlet to Cy5) using (a) Cy5 MFI of total association of mAb‐LNPs or (b) MFI of uptake of mAb‐LNPs in B cells. Receptors shown had significant total association/uptake compared to the isotype control (mIgG1). Statistical analysis was performed using 2‐way ANOVA with Dunnett's test. p > 0.05 is not significant (ns), * indicates p < 0.05, ** indicates p < 0.01, *** indicates p < 0.001 and **** indicates p < 0.0001. Each symbol is representative of mean ratio calculated for a single donor (with technical replicates). Data is mean ± SD (n = 3 individual donors).

These data were used to calculate how efficiently each targeted LNP could induce mScarlet protein expression relative to the total amount of internalised mRNA (Figure 4). For FIP‐mAb‐LNPs that did not exhibit a statistically significant increase in association compared to untreated cells, internalisation efficiency was not calculated (denoted by “#” – Figure 3c). This is because calculating a ratio under conditions where the association is not significantly different from background can yield artificially inflated ratios. Such values do not reflect meaningful biological differences. This expression efficiency provides additional insight into the efficiency of mRNA delivery compared to simply measuring protein expression relative to LNP association. The expression efficiency (relative to association) of CD20 targeted LNPs (∼0.4) was significantly higher than the expression efficiency of CD22 targeted LNPs (∼0.1; Figure 4a). Even more notable, the difference between the expression efficiency relative to total internalised mRNA for CD20 targeted LNPs (∼0.9) and CD22 targeted LNPs (∼0.1) was even higher (Figure 4b). This suggests that despite CD20 targeted LNPs exhibiting lower uptake and internalisation efficiency (∼35%) compared to CD22, the internalised CD20 targeted LNPs are highly effective in translating delivery to protein. This suggests favourable LNP/mRNA post‐internalisation trafficking when delivering through CD20.

CD45 targeted LNPs did not display significant mScarlet expression above untargeted LNPs despite having both significantly higher total association and uptake (Figure 3d). Therefore, expression efficiency could not be accurately calculated and was not included. These data do however suggest that targeting LNPs through CD45 on B cells results in unfavourable LNP/mRNA trafficking following internalisation [15] and poor cytoplasmic delivery and protein expression following 4‐h incubation.

### 2.4 T Cells Show More Efficient Protein Expression with CD3 Targeted LNPs, Despite Having Less Efficient Internalisation

T lymphocytes are broadly responsible for antigen‐presentation recognition, and response, through their effector CD4 and CD8 subclasses, making them critical components of the adaptive immune response [16]. While ex vivo approaches such as CAR T‐cell therapy remain clinically established, their high costs and patient‐specific variability limit broader application [17]. In contrast, direct in vivo targeting of T cells has been more challenging, though advances in nucleic acid therapeutics, particularly mRNA‐loaded LNPs, are rapidly expanding this field. Targeting T cell specific surface receptors now offers a promising strategy for selective mRNA delivery. Among these, CD3 and CD7 represent clinically relevant receptors of high interest, making them compelling candidates for evaluating mAb‐LNP–mediated delivery.

**FIGURE 5:**
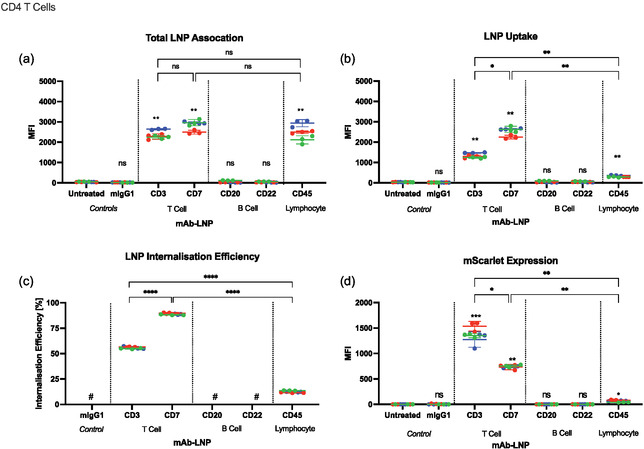
Measuring mAb‐LNP internalisation and delivery in CD4+ T cells after 4 h. SHIP assay outputs result in quantification of (a) total association (MFI), (b) uptake of mAb‐LNPs (MFI) and (c) internalisation efficiency (%) of the receptor when bound by the mAb‐LNP. (d) Represents the mScarlet protein expression. Cy5‐FIP and mScarlet fluorescence were analysed by flow cytometry at 649 and 580 nm, respectively. Uptake of the mAb‐staple sensor through each receptor was measured following the addition of quencher DNA (500 nM). Statistical analysis was performed using 2‐way ANOVA with Dunnett's test. Each mAb‐LNP was compared to the isotype (mIgG1) control. p > 0.05 is not significant (ns), * indicates p < 0.05, ** indicates p < 0.01, *** indicates p < 0.001 and **** indicates p < 0.0001. Significance shown is representative of the mean calculated p value of the donors. # signifies receptors that did not have sufficient association compared to mIgG1 isotype control for internalisation to be calculated. Each colour is representative of a single donor (with technical replicates). Data is mean ± SD (n = 3 individual donors and 3 technical replicates).

In CD4+ T cells, both CD3 targeted (MFI 2387 ± 205) and CD7 targeted LNPs (MFI 2805 ± 252) showed significantly higher association with their target cells after 4 h when compared to untreated cells (MFI 18.2 ± 4.54) (Figure 5a). Similarly, CD45 targeted LNPs (MFI 2516 ± 381), showed strong association with CD4+ T cells. Association between CD3, CD7 and CD45 targeting mAb‐LNPs was not significantly different. Similar trends were observed for CD8+ T cells (Figure S4). This trend in association differs from our previous studies using anti‐CD7 and anti‐CD3 antibodies without the LNP, where mAb targeting CD45 resulted in a ∼sixfold higher association than the mAb targeting CD7 [14]. This suggests that conjugation to LNPs can alter association of the targeting mAb with their receptor. As expected, no significant association was observed for B cell targeted LNPs with either T cell subsets (CD4+ or CD8+), therefore these receptors did not have receptor internalisation efficiency calculated (Figure 5a).

As was observed in B cells, the uptake of the targeted LNPs did not correlate with association (Figure 5b). In CD4+ T cells, CD7 targeted LNPs (MFI 2504 ± 212) showed significantly higher uptake than CD3 targeted LNPs (MFI 1335 ± 114). A similar trend was observed for CD8+ T cells (Figure S4b). We also observed a similar trend for association/expression for CD4+ and CD8+ T cells after 24 h (Figure S5). As was observed in B cells, CD45 targeted LNPs showed significantly lower uptake than the other targeted receptors (MFI 314 ± 39). As per the expression efficiency calculations for B cell targeting, FIP‐mAb‐LNPs that did not exhibit significantly higher association signal than untreated cells were not calculated (Figure 5c). The internalisation efficiency of LNPs targeting CD7 was ∼90% after 4 h (Figure 5c and Figure S4c), which is consistent with our previous studies for internalisation of the anti‐CD7‐mAb [14]. Interestingly, our previous studies showed similarly high levels of internalisation into T cells with both anti‐CD7 and anti‐CD3 antibodies (both > 90%) [14], however conjugation of the anti‐CD3 mAb to the LNP significantly lowers internalisation efficiency to ∼55% (Figure 5c and Figure S4c). This indicates that conjugation of the antibody to the LNPs may interfere with the internalisation of the CD3 receptor. This may be linked to the mechanism of CD3 internalisation, which forms a coreceptor complex with TCR before internalisation [18].

The internalisation efficiency of CD45 targeted LNPs was lower than both CD7 and CD3 targeted LNPs, with only ∼12% internalisation after 4 h. This is consistent with our previous measurements of anti‐CD45 mAb internalisation in T cells [14]. This suggests that the internalisation mechanism of CD45 is not impeded or altered by the presence or size of the LNP. The CD45‐targeted LNPs were also internalised more efficiently in T cells compared to B cells (Figures 3 and 5), which also mirrors what we previously observed with anti‐CD45 mAbs.

**FIGURE 6:**
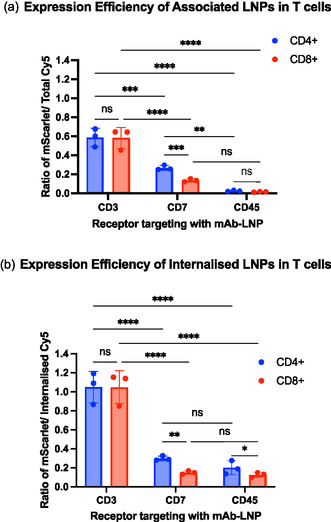
mScarlet expression efficiency of different T cell specific mAb‐LNPs calculated with the total association and internalised Cy5 MFIs. Ratio of MFI (mScarlet to Cy5) using (a) Cy5 MFI of total association of mAb‐LNPs or (b) MFI of uptake of mAb‐LNPs in CD4+ (blue) and CD8+ (red) T cells. Receptors shown had significant total association/ uptake compared to the isotype control (mIgG1). Statistical analysis was performed using 2‐way ANOVA with Dunnett's test. p > 0.05 is not significant (ns), * indicates p < 0.05, ** indicates p < 0.01, *** indicates p < 0.001 and **** indicates p < 0.0001. Each symbol is representative of mean ratio calculated for a single donor (with technical replicates). Data is mean ± SD (n = 3, individual donors).

Despite the high levels of uptake and high internalisation efficiency of CD7 targeted LNPs, mScarlet protein expression in CD4+ T cells was significantly lower for CD7 targeted LNPs (MFI 741 ± 33) than for CD3 targeted (MFI 1388 ± 149). To better understand differences in receptor behaviour we again calculated the expression efficiency (Figure 6). The expression efficiency relative to association for CD3 targeted LNPs (∼0.6) was significantly higher than the expression efficiency of both CD7 and CD45 targeted LNPs (∼0.3 and <0.05, respectively; Figure 6a). The expression efficiency relative to total internalised mRNA for CD3 targeted LNPs was even higher (∼1.0), whilst the expression efficiency of CD7 targeted LNPs (∼0.3) remained the same (Figure 6b). Interestingly the expression efficiency of CD45 targeted LNPs increased when calculated relative to internalised mRNA and was not significantly different to that observed for CD7 targeted LNPs. Also of note, was the different behaviour of the mAb targeted LNPs in CD4+ and CD8+ T cells. While the expression efficiency relative to internalised mRNA for CD3 targeted LNPs was similar for both subsets, for both CD7 and CD45 targeted LNPs, the expression efficiency was lower in CD8+ cells than CD4+ cells.

These data suggest that like CD22 targeted LNPs, while CD7 targeted LNPs are efficiently internalised into T cells, the subsequent processing of the LNP/mRNA is less efficient than the processing of CD3 targeted LNPs. It is interesting to note that the efficiency of expression for CD7 and CD45 targeted LNPs is similar, suggesting the LNP/mRNA may be processed in a similar way.

These data highlight the importance of post‐internalisation trafficking of LNPs and mRNA for subsequent protein expression. Targeting B cell receptors (CD20 and CD22), T cell receptors (CD3 and CD7) and receptors present on both cell types (CD45), all result in highly efficient targeting. However efficient targeting does not always result in efficient protein expression. For the pan T and B cell marker CD45, as was expected very low levels of uptake result in limited or no expression of mScarlet. However more significantly, for CD22 on B cells and and CD7 on T cells, high levels of uptake do not result in efficient expression of the reporter protein. The differences in expression efficiency when targeting different receptors (Figures 4b and 6b) likely results from differences in post‐internalisation trafficking of LNP/mRNA. Variability in receptor‐mediated trafficking pathways may determine whether internalised LNPs reach endosomal compartments that favour mRNA release or instead remain in pathways less conducive to endosomal escape [19, 20]. For example, CD22 is a known endocytic recycling receptor [15] and may favour rapid trafficking between early endosomes and the cell surface, rather than into endosomal compartments that may favour mRNA escape. Conversely, CD20 is internalised slower and less efficiently than dedicated endocytic receptors such as CD22 [21]. Consequently CD20 may lack machinery for rapid receptor recycling, which could extend the dwell time of mRNA‐LNPs in endosomal compartments. This may explain the increase in expression efficiency of mRNA‐LNPs which are internalised by CD20 versus CD22 (Figure 4b).

Additionally, receptor engagement may also influence mRNA translation efficiency through ligand‐induced signalling. Many immune receptors can modulate cellular signalling when bound by antibodies, altering pathways including cellular metabolism and protein synthesis [21, 22, 23, 24, 25, 26, 27, 28, 29, 30, 31, 32, 33, 34, 35]. Antibody binding to CD3 is known to stimulate to T cell activation [18], which may have downstream stimulatory effects on protein machinery resulting in higher protein expression [36, 37]. Such signalling effects could either enhance or dampen the cell's capacity to translate delivered mRNA, contributing to the altered expression efficiencies observed in LNP/mRNA cargoes.

## 3 Conclusions

Cellular interactions of targeted LNPs were investigated to understand the relationship between association, internalisation and mRNA delivery. We chose five model antibodies (anti‐CD3, CD7, CD20, CD22 and CD45) to explore delivery to T and B cell in human PBMCs. The SHIP internalisation assay enabled quantitation of both association and internalisation of the LNPs, with efficiency of mRNA delivery measured by expression of the mScarlet reporter protein. We found that targeting alone and high levels of association with target cells is not a good predictor of subsequent protein expression. Interestingly, while LNP uptake into cells is essential for active mRNA delivery, the amount and efficacy of uptake is also not a good predictor of the efficiency of protein expression. Furthermore, understanding the variabilities occurring between mAb‐LNP cell accumulation/uptake and protein expression levels can provide insights into the post‐internalisation trafficking of mAb‐LNPs, such as efficiency of endosomal escape and cytosolic mRNA processing.

Our data here shows the importance of screening an array of different surface receptors on the target cells to identify the most efficient receptor for inducing protein expression. The SHIP assay is a valuable tool to better understand cellular processing of LNPs and helps facilitate the process of selecting mAbs for LNP targeting that improve LNP accumulation in target cells, to promote favourable trafficking and protein expression to maximise the potential of LNP therapeutics.

## 4 Materials and Methods

### 4.1 mRNA Synthesis

mScarlet mRNA was synthesised using IVT from a PCR template containing a T7 promoter upstream followed by the codon optimised ORF (see Supporting Information for sequence details). All constructs contain a 5′UTR, 3′UTR and 125 polyA tail. All mRNA was transcribed using the HiScribe T7 High Yield RNA Synthesis Kit (New England Biolabs). Capping was performed cotranscriptionally using CleanCap Reagent AG (TriLink Biotechnologies). All uridine was replaced with N 1‐Methylpseudouridine (TriLink Biotechnologies). IVT reaction was treated with DNase to eliminate the template and dsRNA was removed by cellulose clean‐up methods [38]. The final product was purified by sodium acetate precipitation. mRNA was synthesised and donated by the Pouton Lab (Colin Pouton, Monash University).

### 4.2 Oligonucleotides for SHIP Assay

All ssDNA oligonucleotides were ordered from Integrated DNA Technologies (IDT). The oligonucleotide for the click sensor was (1) 5′ Cy5‐ TCA GTT CAG GAC CCT CGG CT ‐NH_3_ 3′. Oligonucleotides were reconstituted in Milli‐Q water to prepare 50 µM stocks. Oligonucleotide (2) (5′ AGC CGA GGG TCC TGA ACT GA‐BHQ2 3′) is the quencher DNA (QP_C_) and was used for quenching in the SHIP assay for both the staple and click sensors.

### 4.3 Anti‐FcNb (TP1107) Expression

pET‐TP1107(Q15TAG) (Addgene #247462) was co‐transformed alongside pEVOL‐pAzF (Addgene #31186) into B‐95.ΔA *E. coli* (Addgene #197933) which expresses the orthogonal machinery for incorporation of azPhe in recognition of UAG codon during protein translation [39]. The B‐95.ΔA *E.* *coli* strain is a unique expression vector where 95 of its original UAG codons have been replaced along with the elimination of release factor 1 (RF‐1) to facilitate improved incorporation efficiency of azPhe [40].

An overnight culture was inoculated into fresh TB media with appropriate antibiotics and grown at 37°C while shaking until the optical density, OD_600_ reached 0.7–1.0. TP1107 nanobody expression was induced by the addition of IPTG (2 mM), L‐arabinose (0.02%) and 4‐Azido‐L‐phenylalanine amino acid (2 mM). Protein expression was continued for a further 12–14 h at 30°C before harvesting the bacteria by centrifugation. Bacterial pellets were harvested by centrifugation (4,000x g, 20 min) and resuspended in Ni‐NTA wash buffer followed by cell lysis using a high‐pressured homogeniser (Avestin Emulsiflex C5).

Upon lysis, cell debris were centrifuged (12,000x g, 30 min) and supernatant collected for purification via an immobilised metal affinity chromatography (IMAC) column. An additional size exclusion chromatography (SEC) was employed to remove non‐specifically bound proteins using Superdex 75 10/300 GL gel filtration column (GE Healthcare). TP1107 nanobody concentration was determined using Nanodrop (Thermo) spectrophotometer at 280 nm.

### 4.4 Anti‐FcNb (TP1107) Conjugation to PEG Lipid

Azide incorporated anti‐FcNb (TP1107) can be directly conjugated onto DBCO‐PEG_2000_‐DSPE through SPAAC chemistry. The conjugation mixture was prepared at molar ratio 2:1, DBCO: Azide. The azide modified/incorporated anti‐FcNb was mixed with DSPE‐PEG_2000_‐DBCO at a 0.5 molar excess and left for 24 h at 37°C. No further purification was required.

### 4.5 LNP Formulation with Anti‐FcNb‐PEG Post Insertion and mAb Conjugation

LNPs were formulated as previously reported with modification [41]. A lipid mixture consisting of SM102, DSPC (Avanti Polar Lipids), cholesterol (Sigma) and 14:0 PEG2000 PE (DMG‐PEG2000) or 18:0 PEG2000 PE (DSPE‐PEG2000) (Avanti Polar Lipids) was prepared in ethanol as a 20 mM stock. The molar composition used was 50:10:38.5:1.5 molar ratio. The lipid solution was mixed by flowing through a micro fluidic mixing device Nanoassemblr (Precision Nanosystems, Vancouver BC) with an aqueous mRNA solution in 10 mM citrate buffer (pH 4) at a 1:3 organic to aqueous volume ratio, 4 ml per min total flow rate. The resulting LNPs were then diluted twice with PBS (pH 7.4) immediately and further dialysed overnight. Then next day, LNPs were filtered through a 0.22 micron filter.

0.5% w/w of DSPE‐PEG2000‐TP1107 mixture was added to prepared LNPs. The reaction was incubated at 4°C for 48 h. The free TP1107 or unreacted DSPE‐PEG2000‐DBCO were removed via Amicon 100 kDa MWCO (Merck) ultrafiltration. A total of five washes (2,000 rpm, 10 min) were completed. Functionalised LNP was prepared by mixing antibodies with anti‐FcNb LNP at a 2:1 ratio and incubated at 4°C overnight.

Verification of mAb capture onto the anti‐FcNb‐LNPs was conducted by protein gel electrophoresis, run under native conditions. Using a pre‐cast, 12% TGX polyacrylamide gel (Bio‐Rad), mAb‐LNP (100 ng protein) were prepared with native page loading buffer. Samples were loaded and the gel was run for 120 min at 100 V in native page buffer.

For Western blot, the gel was transferred onto a nitrocellulose membrane using a Bio‐Rad Trans‐Blot Turbo Transfer System (as per the manufacturer's instructions). Anti‐mouse HRP‐linked antibody was added (1:2000 dilution in blocking buffer) and probed overnight at 4°C. Chemiluminescence substrate was added as per the manufacturer's instructions and the membrane was scanned using the Bio‐Rad ChemiDoc Imager (Bio‐Rad) on the chemiluminescence and colorimetric channels. Channel images were merged for final image.

### 4.6 LNP Characterisation

The size distribution was measured using Nanosight NS300 (Malvern Panalytical). Total mRNA content and encapsulation efficiency were determined by performing a standard Ribogreen (ThermoFisher) assay.

### 4.7 Antibody Conjugation with FIP

Targeting mIgG1 antibody, made in sodium bicarbonate buffer was reacted with a 10x molar excess of NHS‐DBCO for 2 h at 37°C. After 2 h, the reaction was passed through a ZEBA column (ThermoScientific) into PBS, to remove unreacted NHS‐DBCO. The concentration of the DBCO‐antibody was measured by Nanodrop (ThermoScientific). FIP‐azide was then added at a 5x molar excess and incubated overnight at 4°C. The FIP‐antibody was then purified by microcentrifugation using a 50 k Amicon filter to remove free FIP‐azide.

Verification of FIP labelling was done by SDS‐PAGE using a pre‐cast, 12% TGX polyacrylamide gel (Bio‐Rad). 10 µL of the conjugation reactions (and respective controls) were prepared with 5 µL of non‐denaturing SDS‐page loading buffer. The gel was run for 90 min at 120 V in running buffer (25 mM Tris, 250 mM glycine, 0.1% SDS at pH 8.3). Gels were scanned using the Amersham Typhoon Imaging System (Amersham) to detect the presence of the FIP‐Cy5, at 642 nm excitation wavelength.

Antibody‐FIP was measured using Nanodrop and degree of labelling was calculated by Equation (1), as shown below

(1) $$DOL=\frac{A_{\mathrm{dye}}\;\times \;\;\in_{\text{protein}}\;}{\in_{\mathrm{dye}}\left(A_{280}\;-\;CF\;\times \;A_{\mathrm{dye}}\right)}$$

$A_{dye}$ is the absorbance of the dye at its absorbance maximum (for Cy5 is 649 nm). $\in_{dye}$ is the extinction coefficient of the dye at its absorbance maximum. $A_{280}$ is the absorbance of the antibody conjugate at 280 nm. $\;\in_{\text{protein}}$ is the extinction coefficient of the antibody at 280 nm. CF is the empirically obtained correction factor. For Cy5‐FIP, the CF is ∼0.55.

### 4.8 Antibodies Used for LNP SHIP

All mAbs used in this study were purified mouse monoclonal IgG1 anti‐human antibodies. For the FIP functionalisation and LNP binding, antibodies used were: mIgG1 isotype control (clone P3.6.2.8.1) (Invitrogen), CD3 (clone UCHT1) (Invitrogen), CD7 (clone 124‐1D1) (Invitrogen), CD20 (clone MEM‐97) (Invitrogen), CD22 (clone 4KB128) (Invitrogen) and CD45 (clone HI30) (Invitrogen).

### 4.9 Extraction of PBMCs

Primary PBMCs were isolated from donor blood samples by density gradient medium centrifugation using Ficoll–Paque media. Briefly, collected blood is diluted with an equal volume of PBS solution and layered on top of Ficoll–Paque media (Merck, USA) in a 50 mL centrifugation tube. Samples are centrifuged for 30–40 min (400 × g) at 18°C to 20°C (no brake). After centrifugation, plasma (the upper layer) was removed and discarded to reveal the layer of PBMCs which were collected. PBMCs were then diluted with 3× PBS and centrifuged at 400 × g for 10 min (18°C to 20°C) to remove residual media. This step was then repeated. PBMCs were finally resuspended in RPMI media with 2% FBS for use, or FBS with 10% DMSO for cryo‐preservation of the PBMCs.

### 4.10 Internalisation (SHIP) and mScarlet Expression in PBMCs

FIP‐mAbs were generated for mIgG1, CD3, CD7, CD20, CD22 and CD45 mAbs and attached to synthesised LNPs in a 2:1 ratio of LNPs to Fip‐mAb. FIP‐mAb‐LNPs were added to cells at a final concentration of 10 nM (in 70 μL) to 5 × 10^5^ primary PBMCs in a well. Time points for internalisation were taken at 4 h to determine receptor internalisation kinetics for B cells and T cells. Samples were incubated at 37°C and 4°C for 4 h. To stop the reaction, cells were cooled to 4°C. Cells were then washed twice with RPMI/2%FBS and resuspended in RPMI/2%FBS containing the relevant phenotyping panel. Cells were incubated with phenotyping antibodies at 4°C for 30 min. Cells were then washed twice with RPMI /2%FBS and half the volume of the well was transferred to a second well. One of the two wells is resuspended in in PBS and the other in PBS/BHQ2 (solution of 500 nM quencher DNA). Cells are incubated with BHQ2 for 10 mins at 4°C and analysed using flow cytometry. As above, phenotyping antibodies were used to determine PBMC cell populations.

Phenotyping antibodies were used to determine PBMC cell populations. For B cell/ CD4+ T cell/ CD8+ T cell populations, anti‐Human CD4‐BV510 (BioLegend), anti‐Human CD8‐BV785 (BioLegend), anti‐Human CD19‐BV421 (BioLegend), anti‐Human TCR‐FITC (Thermo Fisher) and Zombie‐NIR (Cell Live/Dead stain) (BioLegend) were used.

### 4.11 Calculating Internalisation

Internalisation (%) was calculated via Equation (2) below. All obtained Cy5 MFI values were multiplied by the respective DOL for each mAb‐FIP before further calculations.

P_*n*_ is the geometric MFI at time n without the addition of QP_c_. Q_*n*_ is the geometric MFI at time n after QP_c_ has been added. Q_Eff_ is the quenching efficiency. Quenching efficiency is given by Equation (3) below where the same annotations apply but samples are incubated at 4°C

(2) $$\%\;\text{Internalised}\;\left[37{}^{\circ}C\right]=\left[1-\left(\frac{Pn-Qn}{Pn*(1-\mathrm{Qeff})}\right)\right]*100$$

(3) $$\text{Quenching efficiency ratio}\;\left[4{}^{\circ}C\right]=\left[1-\left(\frac{Pn}{Qn}\right)\right]$$

### 4.12 Calculating Expression Efficiency

Protein expression efficiency ratio was calculated using the MFI of the Cy5 (representing total association or internalised signal) and the MFI of the mScarlet (protein expression). The final ratio is used to indicate how efficiently association or uptake is relative to the generated mScarlet signal. MFI of mScarlet is the MFI generated by the expression of mScarlet for a given mAb‐LNP. MFI of Cy5 is the Cy5 MFI generated by the total association of the mAb‐LNPs or the uptake of mAb‐LNPs (depending on assay used).

(4) $$\text{mScarlet expression efficiency ratio}=\left[\left(\frac{MFI\;of\;\text{mScarlet}\;}{MFI\;of\;Cy5\;}\right)\right]$$

### 4.13 Flow Cytometry

Flow cytometry was performed with a Cytek Aurora 5 laser cytometer and data were analysed using FlowJo (BD Biosciences, version 10.10.0, Tree Star, Oregon, USA).

### 4.14 Statistical Analysis

Data are presented as mean ± standard deviation based on the data obtained from at least *n* = 3 independent experiments or wells, as indicated in the figure caption. Statistical significance was determined using GraphPad Prism 9.0 and stated in each figure legend.

## Funding

This work was supported by the Australian Research Council (DP250102391 and DP250101727).

## Ethics Approval Statement

Healthy donors aged between 18 and 50 years old in both sexes were recruited voluntarily after the invitation to participate. The ethics is approved by Monash University Human Research Ethics Committee, application ID 37 405.

## Conflicts of Interest

The authors declare no conflicts of interest.

## Supporting information

Supplementary Material

## Data Availability

The data that support the findings of this study are available from the corresponding author upon reasonable request.
