# Supplementary material for: Entry Without Expression: Internalisation Does Not Predict mRNA Translation for Targeted Lipid Nanoparticles
Source: Small Sci. 2026 May 21;6(5):e70313. doi: 10.1002/smsc.70313 (PMC13248845; doi:10.1002/smsc.70313)
Supplement: Supplementary file 1 — Supplementary Material [file SMSC-6-e70313-s001.pdf]

Entry without expression: Internalization does not predict mRNA translation for targeted LNPs

Cameron H. Smyth[#], Lara M. Mollé[#], Victoria McLeod, Moore Z. Chen, Angus P.R. Johnston*

# Authors contributed equally

C.H. Smyth, L.M. Mollé, V. McLeod, M.Z. Chen, A.P.R. Johnston
Drug Delivery, Disposition and Dynamics, Monash Institute of Pharmaceutical Sciences, Monash University, Parkville, Victoria, Australia
E-mail: angus.johnston@monash.edu

Supplementary Data

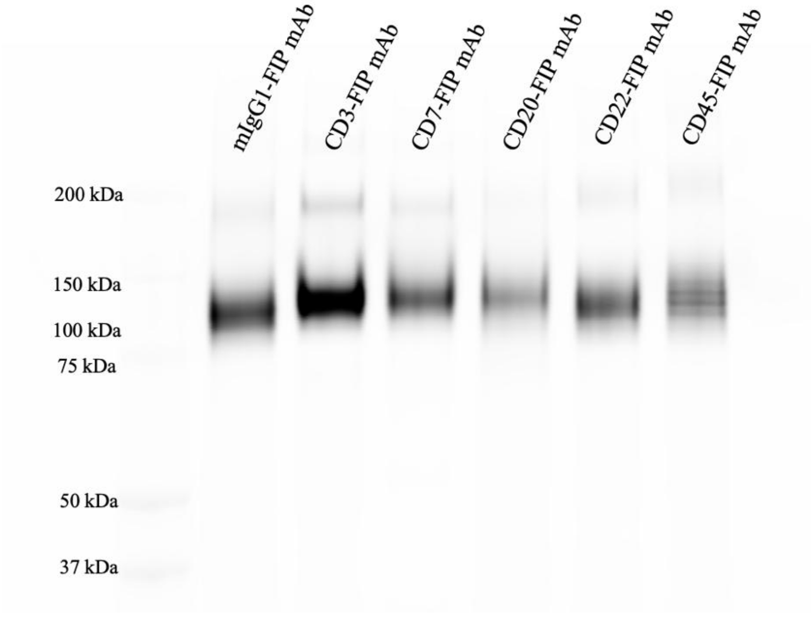

Figure S1. Click conjugation of FIP-Cy5 onto antibodies. *Click conjugation of FIP-Cy5 onto the mAbs of interest is confirmed by fluorescent imaging (680nm) following SDS-PAGE analysis. Degree of labelling determined for all FIP-mAbs and applied when quantifying internalization.*

**Degree of Labelling of Functionalized FIP-mAbs**	
**Antibody**	**DOL**
mIgG1	2.27
CD3	2.38
CD7	2.06
CD20	2.84
CD22	2.54
CD45	2.83

Table *S1*. Summary of degree of labelling of functionalized FIP-mAbs.

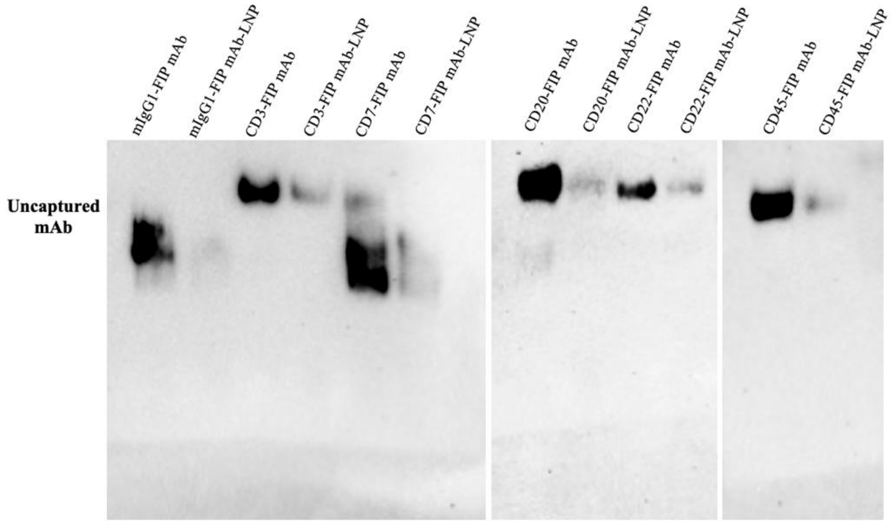

Figure S2. FIP-mAbs can be efficiently functionalized onto LNP with minimal free mAb present. *The incubation period was 24 hours at 37℃. Protein gel was run under native conditions and followed by western blot detecting the anti-human antibodies raised in mice. Only free FIP-mAbs can enter the gel, when FIP-mAbs are attached to the LNP they remain in the well. The absence of a band indicates there is no free FIP-mAb present in solution.*

	Characterisation data of formulated lipid nanoparticles			
**LNP**	Size (nm)	SD	Encapsulation Efficiency (%)	Stock concentration (ng/μL of encapsulated mRNA)
Untargeted LNP	101	3.1	98	10
mAb-mIgG1-TP1107-LNP	108	8.3	94	8.9

Table *S2*. Summary of the characterisation data of formulated untargeted and targeted LNPs measured by NTA. This includes hydrodynamic diameter (nm), standard deviation, encapsulation efficiency of the mRNA (%) and the stock concentration of the LNPs for cell dosing (ng/μL of encapsulated mRNA). The stock concentration of mAb-LNPs is the same for each antibody tested, as they are all formulated from the same base formulation.

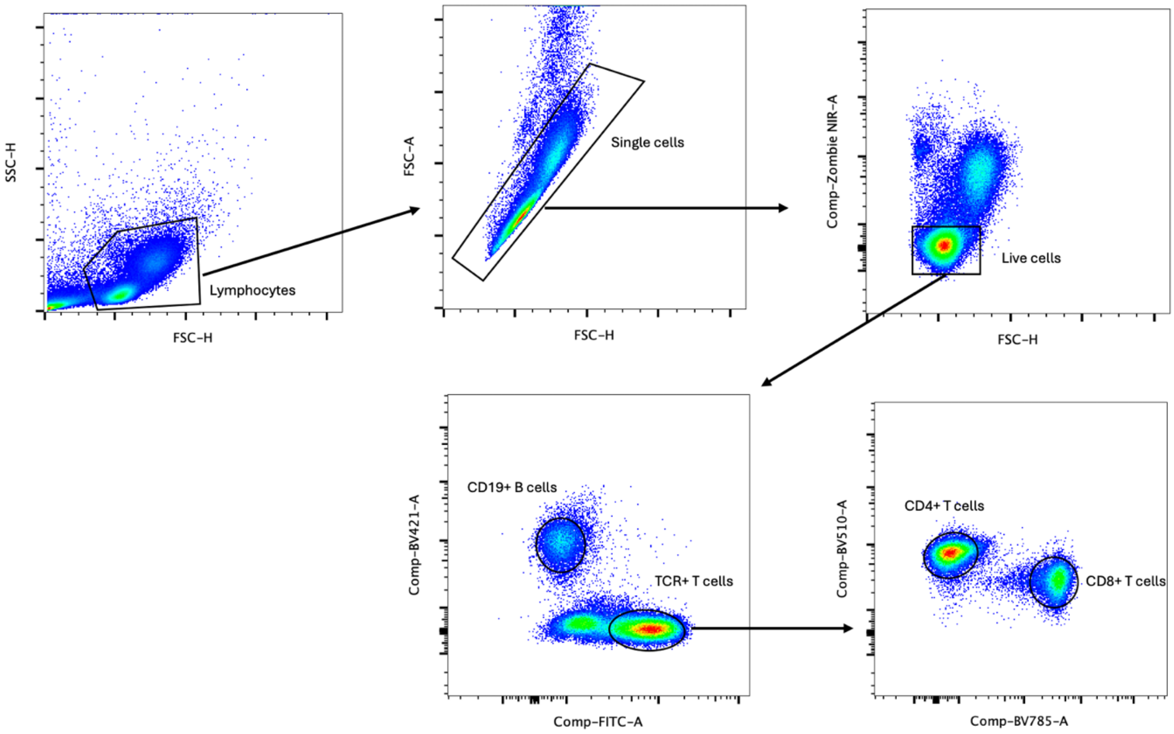

Figure S3. ***Gating strategy of identifying subpopulation in purified human PBMC.*** *Purified human PBMC were stained with an antibody cocktail (Live and Dead dye, antiTCR, antiCD19, antiCD4, antiCD8a). Lymphocytes were selected first then subphenotyped into different groups as shown in the figure.*

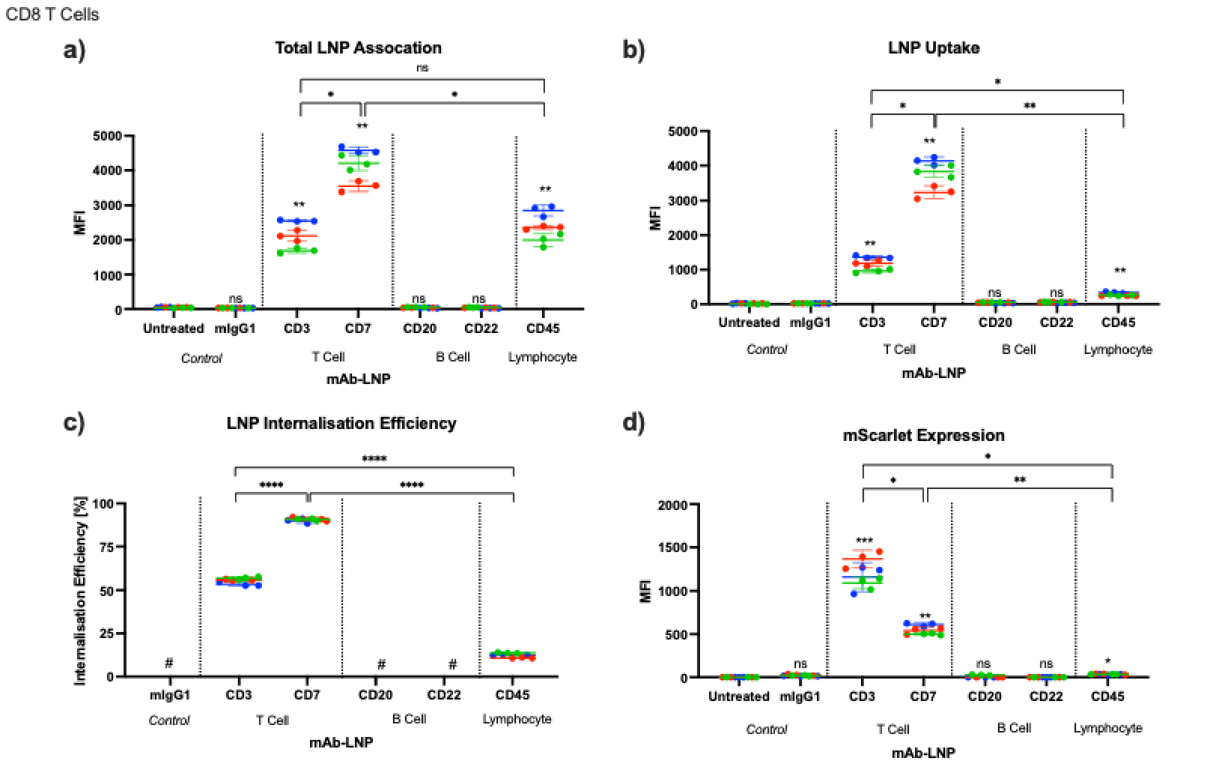

Figure S4: ***Measuring mAb-LNP internalisation and delivery in CD8+ T cells after 4 hours.*** *SHIP assay outputs result in quantification of a) Total Association (MFI), b) Uptake of mAb-LNPs (MFI) and c) Internalisation efficiency (%) of the receptor when bound by the mAb-LNP. d) Represents the mScarlet protein expression. Cy5-FIP and mScarlet fluorescence were analysed by flow cytometry at 649 nm and 580 nm, respectively. Uptake of the mAb-staple sensor through each receptor was measured following the addition of quencher DNA (500nM). Statistical analysis was performed using 2-way ANOVA with Dunnett's test. Each mAb-LNP was compared to the untreated cell control. p>0.05 is not significant (ns), * indicates p<0.05, ** indicates p<0.01, *** indicates p<0.001 and **** indicates p<0.0001. Significance shown is representative of the mean calculated p value of the donors. # signifies receptors that did not have sufficient association compared to mIgG1 isotype control for internalisation to be calculated. Each colour is representative of a single donor (with technical replicates). Data is mean ± SD (n=3 individual donors and 3 technical replicates).*

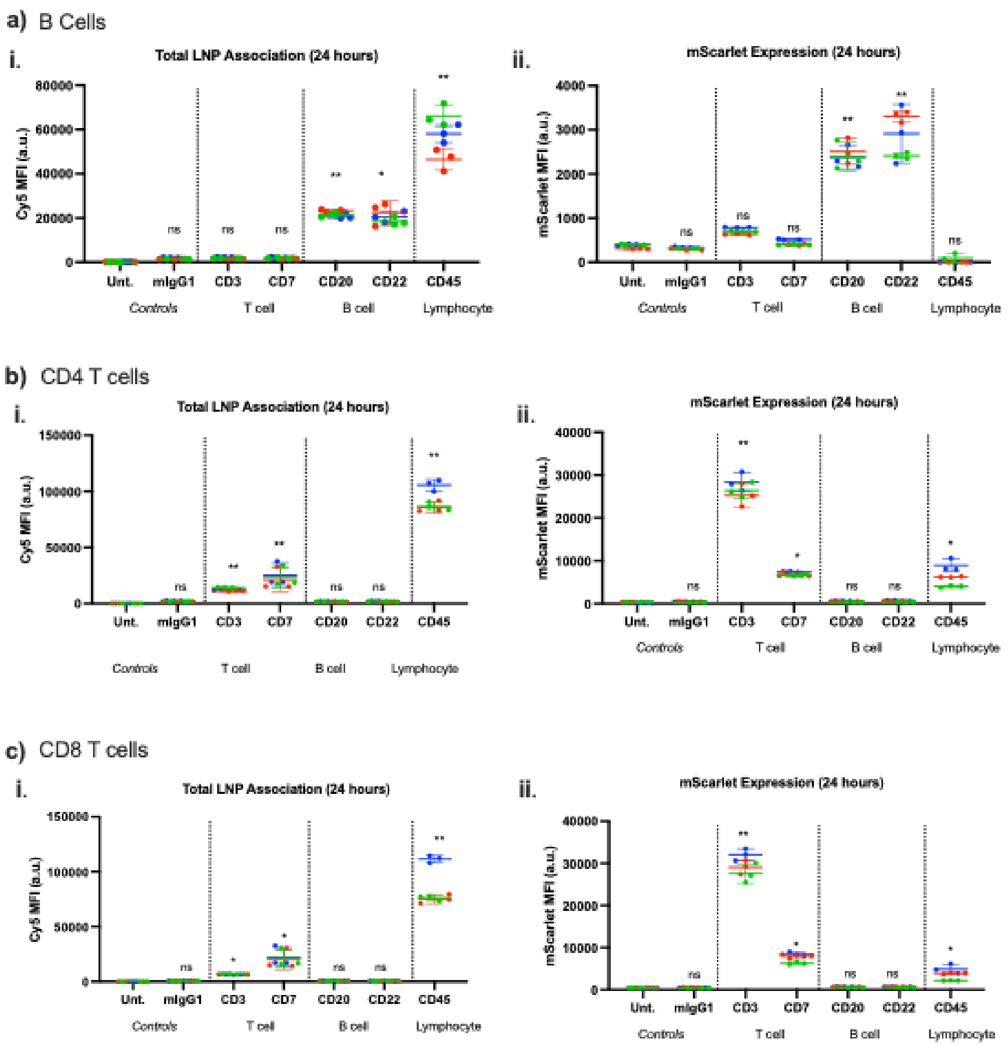

Figure S5: ***mAb-LNP association and expression in PBMC after 24 hours.*** *LNPs targeted with mAb and encapsulated with mScarlet mRNA and a Cy5-conjugated oligonucleotide were incubated with PBMC for 24 hours. Cells were phenotyped into a) B cells, b) CD4+ T cells and, c) CD8+ T cells. Assay outputs result in quantification of i) Total Association (Cy5 MFI) and ii) mScarlet protein expression (mScarlet MFI). Cy5 and mScarlet fluorescence were analysed by flow cytometry at 649 nm and 580 nm, respectively. Statistical analysis was performed using 2-way ANOVA with Dunnett's test. Each mAb-LNP was compared to the untreated (unt.) cell control. p>0.05 is not significant (ns), * indicates p<0.05, ** indicates p<0.01, *** indicates p<0.001 and **** indicates p<0.0001. Significance shown is representative of the mean calculated p value of the donors. Each colour is representative of a single donor (with technical replicates). Data is mean ± SD (n=3 individual donors and 3 technical replicates).*

>mScarlet mRNA sequence

GACCCGGGAUAAGCCGCCACCAUGGUGAGCAAGGGCGAGGCGGUCAUCAAGGAGUUCAUG
CGCUUCAAGGUCCACAUGGAGGGCAGCAUGAACGGGCACGAGUUCGAGAUCGAGGGCGAG
GGGGAGGGCCGGCCGUACGAGGGGACGCAGACGGCCAAGCUGAAGGUCACGAAGGGCGGC
CCGCUGCCGUUCAGCUGGGACAUCCUGAGCCCCCAGUUCAUGUACGGGAGCCGGGCCUUCA
UCAAGCACCCCGCCGACAUCCCGGACUACUACAAGCAGAGCUUCCCGGAGGGCUUCAAGUG
GGAGCGGGUCAUGAACUUCGAGGACGGGGGGGCGGUGACGGUGACGCAGGACACCAGCCU
GGAGGACGGGACGCUGAUCUACAAGGUCAAGCUCCGCGGCACGAACUUCCCGCCCGACGGC
CCGGUCAUGCAGAAGAAGACGAUGGGGUGGGAGGCGAGCACGGAGCGCCUGUACCCCGAG
GACGGGGUGCUGAAGGGCGACAUCAAGAUGGCGCUGCGGCUCAAGGACGGGGGCCGGUAC
CUCGCCGACUUCAAGACGACCUACAAGGCGAAGAAGCCGGUGCAGAUGCCGGGGGCCUAC
AACGUGGACCGCAAGCUCGACAUCACGAGCCACAACGAGGACUACACGGUGGUGGAGCAG
UACGAGCGCAGCGAGGGGCGGCACAGCACCGGGGGGAUGGACGAGCUGUACAAGGGCAGC
GGCGCGACGAACUUCAGCCUGCUGAAGCAGGCCGGCGACGUGGAGGAGAACCCCGGGCCG
AUGGUCUUCACCCUGGAGGACUUCGUGGGCGACUGGCGGCAGACGGCGGGGUACAACCUG
GACCAGGUCCUGGAGCAGGGCGGCGUCAGCAGCCUGUUCCAGAACCUGGGGGUGAGCGUC
ACGCCCAUCCAGCGCAUCGUCCUCAGCGGGGAGAACGGCCUGAAGAUCGACAUCCACGUGA
UCAUCCCCUACGAGGGCCUCAGCGGGGACCAGAUGGGGCAGAUCGAGAAGAUCUUCAAGG
UGGUGUACCCGGUCGACGACCACCACUUCAAGGUGAUCCUCCACUACGGCACGCUGGUGAU
CGACGGCGUCACCCCCAACAUGAUCGACUACUUCGGGCGGCCCUACGAGGGGAUCGCGGUG
UUCGACGGCAAGAAGAUCACCGUCACGGGGACCCUCUGGAACGGGAACAAGAUCAUCGAC
GAGCGCCUCAUCAACCCGGACGGGAGCCUCCUCUUCCGGGUGACGAUCAACGGGGUCACCG
GGUGGCGGCUGUGCGAGCGCAUCCUCGCGUGAUAGCCCGGGGACUCUAGAGUCGGGGCGG
CCGGCCGCUUCGAGCAGACAUGGUUUAAACGAU

>TP1107Q15 DNA sequence

ATGGGTCAAGTGCAACTTGTAGAAAGTGGTGGGGGCCTTGTTTAGCCTGGGGGGTCTCTT
CGTTTATCGTGTGCGGCTTCCGGTTTCACCTTCTCCGATACATGGATGAATTGGGTACGTC
AGGCCCCCGGGAAAGGCTTATATTGGATTTCGGCGATTAACCCGGATGGGGGCAATACA
GCTTACGCTGACTCAGTAAAGGGCCGTTTCACTATCAGCCGCGATAACGCAAAAAACAT
GGTCTACTTGCAAATGGACAATCTTCGTCCGGAGGACACGGCGATGTACTACTGCGCCAA
GGGCTGGGTCCGCTTACCAGATCCGGACCTGGTACGCGGACAAGGCACGCAAGTGACAG
TTTCCTCCGAAAATCTTTACTTCCAGGGCCATCATCATCATCACCATTGA
